# Three-Dimensional Bioreactor Technologies for the Cocultivation of Human Mesenchymal Stem/Stromal Cells and Beta Cells

**DOI:** 10.1155/2018/2547098

**Published:** 2018-03-14

**Authors:** Florian Petry, Tobias Weidner, Peter Czermak, Denise Salzig

**Affiliations:** ^1^Institute of Bioprocess Engineering and Pharmaceutical Technology, University of Applied Sciences Mittelhessen, Wiesenstraße 14, 35390 Giessen, Germany; ^2^Department of Chemical Engineering, Kansas State University, Manhattan, KS, USA; ^3^Project Group Bioresources, Fraunhofer Institute for Molecular Biology and Applied Ecology (IME), Winchesterstr. 3, 35394 Giessen, Germany

## Abstract

Diabetes is a prominent health problem caused by the failure of pancreatic beta cells. One therapeutic approach is the transplantation of functional beta cells, but it is difficult to generate sufficient beta cells *in vitro* and to ensure these cells remain viable at the transplantation site. Beta cells suffer from hypoxia, undergo apoptosis, or are attacked by the host immune system. Human mesenchymal stem/stromal cells (hMSCs) can improve the functionality and survival of beta cells *in vivo* and *in vitro* due to direct cell contact or the secretion of trophic factors. Current cocultivation concepts with beta cells are simple and cannot exploit the favorable properties of hMSCs. Beta cells need a three-dimensional (3D) environment to function correctly, and the cocultivation setup is therefore more complex. This review discusses 3D cultivation forms (aggregates, capsules, and carriers) for hMSCs and beta cells and strategies for large-scale cultivation. We have determined process parameters that must be balanced and considered for the cocultivation of hMSCs and beta cells, and we present several bioreactor setups that are suitable for such an innovative cocultivation approach. Bioprocess engineering of the cocultivation processes is necessary to achieve successful beta cell therapy.

## 1. Introduction

There are an estimated 422 million diabetes patients worldwide, reflecting the growing prevalence of obesity, inactivity, stress, and smoking [1]. The clinical factor that ultimately links all diabetes patients is the failure of pancreatic beta cells. Most patients suffer from type-2 diabetes, which is initiated by insulin resistance in muscle and adipose tissue often beginning years before diabetes is diagnosed [2]. Insulin resistance leads to hyperinsulinemia, which combined with glucose toxicity enhances the dysfunction of the insulin-producing beta cells [3]. In contrast, type-1 diabetes is innate and characterized by the selective autoimmune destruction of beta cells. Diabetes patients must control their blood glucose level very strictly and many need to inject insulin on a regular basis. Insulin injections are a significant burden for the patients and cannot imitate the precise control of blood glucose by functional beta cells, leading to acute and/or chronic complications. Therapeutic options that retain functional beta cell mass or prevent/reverse the degeneration of beta cell function would therefore be highly beneficial. Replacement strategies include the transplantation of whole human/porcine pancreatic islets, beta cell pseudoislets, or the application of islet progenitors derived from induced pluripotent stem cells (iPSCs) [4, 5]. Several clinical phase I/II trials have demonstrated the safety and efficacy of transplanted islets and beta cell grafts [6] (https://www.clinicaltrials.gov/; condition/disease: diabetes, other terms: beta cells, islets, biological; August 2, 2017, 15:13).

**Figure 1:**
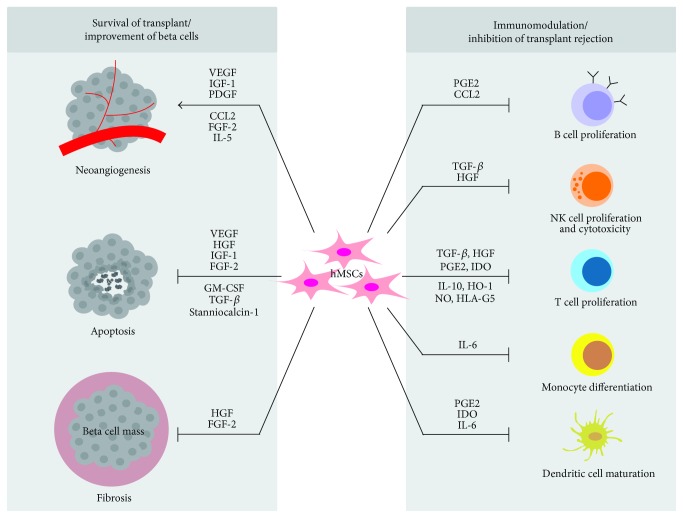
Therapeutic effect of human mesenchymal stem/stromal cells (hMSCs) in the context of beta cell engraftment. Human MSCs modulate the host immune systems, for example, by secreting various trophic factors. Therefore, they prevent rejection of allogenic beta cell grafts and improve the survival of the graft by promoting neoangiogenesis at the transplant site and prevent apoptosis and fibrosis. ┤ inhibition, → improvement. Abbreviations: VEGF: vascular endothelial growth factor; IGF-1: insulin-like growth factor 1; PDGF: platelet-derived growth factor; CCL2: monocyte chemoattractant protein-1; FGF-2: basic fibroblast growth factor; IL-5/6/10: interleukins 5, 6, and 10; HGF: hepatocyte growth factor; GM-CSF: granulocyte macrophage colony-stimulating factor; TGF-*β*: transforming growth factor beta; PGE2: prostaglandin E2; IDO: indoleamine 2,3-dioxygenase; HO-1: heme oxygenase 1; NO: nitrogen monoxide; HLA-G5: human leukocyte antigen-G5.

Most islet/beta cell replacement approaches face a number of challenges. First, there must be a guaranteed supply of suitable islets or beta cells. Like other transplantation types, the amount of donor tissue is often limited. One solution is an efficient expansion protocol for islets or beta cells, and another is the generation of islets from iPSCs or other stem cells. Although this addresses the scarcity of the resource, it does not solve the issue that beta cells in the transplanted grafts tend to undergo apoptosis due to the disrupted connection with the extracellular matrix (ECM) and inhospitable conditions at the transplantation site (e.g., hypoxia or missing vascularization). A further barrier for the long-term survival of transplanted cells is graft-versus-host disease (GVHD), fibrotic overgrowth due to the host inflammatory response, and in diabetic patients a general loss of immune system control. Cell death at the transplantation site can be addressed by helping beta cells to withstand the shock after transplantation. One such strategy for beta cells is cocultivation or cotransplantation with human mesenchymal stem/stromal cells (hMSCs), which play a key role in regenerative medicine and tissue engineering. The ability of hMSCs to modulate and suppress the immune system [7–12] could be particularly advantageous for the coapplication of beta cells (Figure 1). This ability is based on the secretion of large quantities of cytokines such as tumor necrosis factor alpha (TNF*α*) and stanniocalcin-1 (STC-1) [13–15], communication with injured cells, and the resulting strong induction of downstream genes [14, 16, 17]. Lee et al. [18] observed that the administration of hMSCs (i.v.) to mice with a myocardial infarction improved cardiac function due to the expression of TNF*α*-stimulated gene 6 protein (TSG-6). The anti-inflammatory effect occurred when the injected hMSCs were kept as microemboli in the lung after 12–24 h. Until this time, half of the hMSCs had already died. Therefore, the culture conditions and pretreatment of the cells could be important to enhance and accelerate the therapeutic effect [13]. In addition to TNF*α* and STC-1, hMSCs secrete other cytokines such as vascular endothelial growth factor (VEGF), hypoxia-inducible factor 1-alpha (HIF-1*α*), and signaling molecules [19] which can strengthen beta cells and help them to survive at the transplantation site (Figure 1). To use the beneficial MSC effect in clinical practice, a 3D, high-cell-mass cocultivation of beta cells and MSCs is needed, because large numbers of functional cells are required for therapy (10^6^–10^10^ cells per dose) [20]. Beta cells in particular lose most of their functionality in 2D cultures, because cultivation processes based on traditional monolayers in static tissue vessels do not mirror the complexity of the original tissue [21–23]. Monolayer cultures cannot provide mechanical stimulation and have limited cell-to-cell interactions and communication with ECM [21–24]. As an escape strategy, beta cells (and adherent eukaryotic cells in general) tend to form agglomerates *in vitro* to reconstitute the unique 3D environment in the body. Therefore, cell culture and tissue engineering should mimic the natural environment; that is, we must move away from flat monocultures and towards 3D cocultures. This opens the door for innovative bioreactor systems that enable the high-throughput manufacturing of cell agglomerates, spheroids, and organoids up to fully developed organs. Bioreactors create the microenvironment of the cells and offer the possibility to directly monitor and control it.

Here, we describe potential strategies to generate 3D cultures of beta cells, hMSCs, and also cocultures of both cell types. Based on what is now known about hMSC and beta cell cultivation in certain bioreactor systems, we discuss the challenges and parameters for cocultivation in a bioreactor, as well as bioreactor configurations suitable for the cocultivation of hMSCs and beta cells for high-cell-mass expansion or improved cell functionality.

## 2. Three-Dimensional Cultivation Forms for hMSCs and Beta Cells

A prerequisite for 3D bioreactor cultivation is the creation of a 3D cellular microenvironment. For hMSCs and beta cells, several cultivation forms are applicable and these are discussed below.

### 2.1. Cell Aggregation and Spheroid Formation

Beta cells originate from cell islets in the pancreas (islets of Langerhans) and have a strong tendency to agglomerate and form contacts via gap junctions and cell-adhesion molecules, such as cadherins [25]. Therefore, the reconstitution of the natural microenvironment in the form of spheroids helps beta cells to achieve high viability, rapid proliferation, a stable cell fate, a sufficient nutrient supply, and especially their function in glucose homeostasis [25, 26]. Beta cells spontaneously aggregate when cultivated *in vitro* on a low-attachment surface with gentle movement. Amin et al. [27] produced beta cell spheroids in customized micromolds (384-well format) in a standard cell culture plate, achieving an output of 200,000 uniform spheroids with a diameter < 100 *μ*m. The 2D- and 3D-expanded genetically modified beta cell line INS-1832/13 was superior in performance to reaggregated human islets, as indicated by the higher glucose-dependent stimulation index (SI) value for insulin. The cost of 3D cell cultures produced in this manner was not significantly higher than the cost of 2D cultures, and the cells can be expanded using standard reagents and protocols. It is important to control the size of the aggregates or spheroids because mass transport limitations cause aggregates larger than 200 *μ*m in diameter to be undersupplied *in vitro* [26, 28]. Beta cells have a high oxygen demand, and oxygen transport within the aggregates occurs only by diffusion. Furthermore, hypoxic conditions are present at the transplantation site. The spheroids strongly promote angiogenesis and vasculogenesis, overcoming the limitations of mass transfer within cell aggregates at the transplantation site [29].

MSCs are usually expanded in 2D cultures in plastic vessels without losing their stem cell fate, but researchers have realized that such an environment alters the native phenotype of the MSCs [30–32]. Similar to beta cells, MSCs form clusters of 500–10,000 cells that define their own microenvironment, thus preserving and defining the MSC phenotype and the inherent properties of the cells [13, 21, 32, 33]. Apoptosis in MSC spheroids depends on the spheroid size, cell number, and cultivation time. During the initial phase of spheroid formation, apoptosis/necrosis was rarely detected for agglomerates of 25,000 cells after 3 d [34]. Apoptosis-regulating molecules like caspases, Notch, or interleukin-1 are triggered by the agglomeration and induce the production of TSG-6, STC-1, and other therapeutic molecules [13, 34–36]. Bartosh et al. [13] showed that hMSCs agglomerated using the hanging drop method express and secrete higher levels of TSG-6 than 2D-cultured hMSCs. Baraniak et al. [37] produced uniform cell spheroids by forced aggregation in a scaffold-free system. They used murine MSCs and seeded the cells at different densities in AggreWell™ six-well inserts to generate agglomerates by centrifugation. The self-assembly of cell aggregates *in vitro* and consequently the building of bridges between cells are facilitated by the mutual interaction of cadherin and integrin with proteins of the ECM [32, 38]. The type of cadherin depends on the origin of the MSCs: in bone marrow-derived MSCs (BM MSCs), N-cadherin and cadherin-11 are preferentially expressed [32, 39], whereas E-cadherin is expressed most strongly in umbilical cord MSCs (UC MSCs) [32, 40]. The appearance of MSC aggregates is not constant and homogeneous: MSC spheroids undergo current changes and realignments based on their networking with the ECM, reflecting the levels of cadherin/integrin and cortical tension [32]. This behavior results in the development of rounded cells with low intracellular tension within cell aggregates and spreading cells with high intracellular tension on the outside [32, 41, 42]. The permanent rearrangement results in the compaction of the cell spheroids *in vitro*, as indicated by the decreasing diameter (from 630 to 350 *μ*m) over 21 days [13, 32, 43].

Cellular behavior during the cocultivation of hMSCs and beta cells is even more dynamic, and the optimal conditions are not yet determined. MSCs can be cocultured directly with islets/beta cells, indirectly as feeder cells, or in a mixed mode. All these modes are beneficial for the islet/beta cells indicating that MSC-secreted factors are not enough to improve islet/beta cell quality, and direct cell-cell contact is also needed [44]. In direct coculture with intact islets, hMSCs attach mainly to the outside of the islets and did not penetrate them. The MSCs started to differentiate into progenitors of insulin-releasing cells. The indirect cocultivation of islets and MSCs had a positive effect on islet cell viability compared to monocultured intact islets and direct cocultured islets with MSCs. The survival rate of indirect cocultured islet was 60% after four weeks compared to 10% for monocultured and direct cocultured islets [45]. Long-term insulin immunostaining revealed high insulin expression in cocultured spheroids but little insulin in direct cocultured spheroids and monocultured islets. The expression of E-cadherin (a protein that may guide islet architecture and promote insulin secretion) was also higher. The glucose responsiveness (SI values) for the cocultured spheroids was higher than that for the monocultured islets [46]. Interesting phenomena occur when dispersed islet cells and MSCs are mixed and cultivated together. They initially formed an aggregate with an even distribution of both cell types, but after 3 days, the two populations started to separate. After 14 days, the separation of MSCs and islet cells was complete and all that remained was aggregated islets and spheroids. This demixing seemed to be cell-type dependent because a MSC-hepatocyte coculture remained intermixed [46]. It is not clear why beta cells show this behavior and whether it is reproducible with other MSC types or in other cultivation setups, but this result highlights the complexity of coculture setups and the importance of a tight control of the cellular environment. Environmental control can be achieved in bioreactors, but aggregated cells have the drawback that they are not protected against hydrodynamic stress in the bioreactor. Therefore, other cultivation forms are more advantageous for the cocultivation of hMSCs and beta cells in a bioreactor.

### 2.2. Cell Growth on Carriers

**Figure 2:**
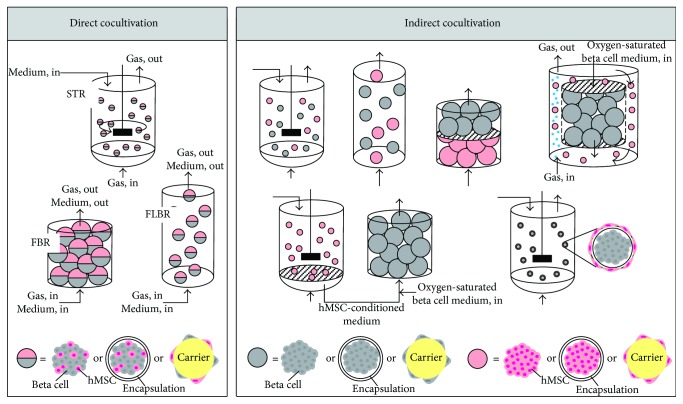
Bioreactor concepts for the cocultivation of human mesenchymal stem/stromal cells (hMSCs) and beta cells. Beta cells and hMSCs can be cocultivated using either a direct mode or an indirect mode. Therefore, the cells can be present as aggregates or as encapsulated cells or attached to a carrier. In direct cocultivation, one medium and one aeration rate must be chosen for both cells, whereas indirect cocultivation allows each cell type to be supplied with a cell-specific medium and aeration rate. Abbreviations: STR: stirred tank reactor; FBR: fixed-bed reactor; FLBR: fluidized-bed reactor.

One suitable form of cultivation widely used in bioreactor systems is cell growth on carriers of different sizes, that is, microcarriers of 100–300 *μ*m and macrocarriers of 0.6–5 mm. Carrier-based cultures are considered as a form of 3D cultivation, but nevertheless, the cells grow on flat or round substrates and the positive effect of aggregated cells is lost, so the term “pseudo-3D” is more appropriate. Microcarriers have been used for decades and come in porous and nonporous types. Porous microcarriers probably mimic 3D cell-cell interactions more accurately than nonporous microcarriers, but the surface of the latter can be modified to achieve some environmental influence, for example, by coating with collagen to enhance cell attachment or with laminin or vitronectin to change the surface charge [47]. All microcarrier types facilitate cell attachment and proliferation, but it remains a challenge to achieve high cell harvest yields without any harmful impact on the cells [48]. Therefore, the aim is to find a balance between efficient attachment/growth and high yields when the cells are harvested. Microcarriers offer a simple and efficient way to expand hMSCs and achieve clinically relevant numbers of cells with the required characteristics [19]. These psuedo-3D processes do not provide all the beneficial properties of 3D cultures but can generate hMSCs that show high vitality and a strong capacity to differentiate. Several microcarrier types have been shown to work for hMSC expansion, as reviewed in detail elsewhere [49, 50]. Islet and beta cells have also been grown on microcarriers since the late 1980s [51]. Single human islets cells were successfully grown on nonporous Cytodex-1 microcarriers for up to 8 days and showed stable insulin secretion [52]. Dispersed islet cells grown on macroporous CultiSpher-S carriers were highly viable and metabolically active, and the SI was ~60% of that achieved by intact bovine islets within alginate microcapsules [53]. The investigation of a beta cell line growing on both carrier types confirmed efficient cell growth and improved insulin secretion compared to single cells and pseudoislets [54]. These studies showed that microcarriers not only facilitate the growth of hMSCs and/or beta cells but also improve their functionality. Microcarriers enable all forms of cocultivation (direct, indirect, and mixed) of hMSCs and beta cells. In direct cocultivation, both cell types are together added to the bioreactor and should distribute homogenously to the microcarriers. The indirect cocultivation includes two separate inoculation processes. First, each cell type is seeded to the suitable microcarrier type, and second, the two batches are combined. The advantage of this setup is the utilization of different microcarrier types adjusted to the specific cell requirements. The disadvantage is the fact that the cells cannot be separated completely. The cells are able to move/migrate to different microcarriers called bead-to-bead transfer [19, 50]. If a strict separation is needed, compartmentation has to be carried out in the cultivation setup, for example, by sieves/membranes (Figure 2). For both cocultivation strategies, the formation of large microcarrier agglomerations is unwanted, due to mass transfer limitations and a more difficult enzymatic harvest of the cells within the agglomerates. This problem of cell harvest must be solved and balanced for two cell types with different detachment behaviors and sensitivities to harvest conditions. For differentiation setups, a targeted clustering of cell-coated microcarriers to a kind of macrotissue can be done [55]. Here, mass transfer has to be balanced as well, but cell harvest must not be considered.

### 2.3. Encapsulation

Nonporous microcarriers cannot protect cells or provide a realistic 3D environment, whereas porous carriers tend to be more 3D-like, thus protecting cells by reducing shear stress. Encapsulation can do even more: cells are protected not only against hydrodynamic shear forces but also against the host immune system at the transplantation site. Encapsulation also maintains or improves cell functionality [56]. The encapsulation material strongly influences the cells and should ideally mimic the geometry, chemistry, and signaling environment of the natural ECM. The most common encapsulation materials are natural polymers, due to their biocompatibility and the mild polymerization conditions [56]. The current materials used in tissue engineering have been reviewed [22]. It is important to guarantee the exchange of secreted molecules, the exclusion of immune system cells/components, the secure entrapment of the encapsulated cells, and a sufficient nutrient supply. The permeability and concentration of the matrix material influence the rate of diffusion, whereas the capsule size and additional membrane coatings determine whether nutrients are transported through the matrix to reach the cells. These factors must be adjusted depending on the nutrients required by the encapsulated cells. In the case of encapsulated spheroids, the major mass transport limitation is determined by the spheroid itself and the matrix permeability can be ignored [56, 57].

In the vast majority of islet transplantation and cultivation approaches, alginate has been used as the encapsulation material [58]. Alginate modifications that cause less foreign-body reactivity have promoted interest in alginate microencapsulation [59]. Alginate encapsulation has a positive impact on beta cells and hMSCs. For example, the encapsulation of pseudoislets in alginate or collagen-alginate increased cell viability and improved functionality compared to nonencapsulated islets *in vitro* and *in vivo* [26]. Furthermore, hMSCs have also been successfully cultivated in alginate capsules, often to achieve a desirable form of differentiation [60]. The mechanical and surface properties of the encapsulation material influence the organization, function, and proliferation of hMSCs, as well as their secretion of trophic factors and their multilineage potential [61, 62]. The maintenance of undifferentiated and highly proliferative hMSCs in alginate capsules is important for their use as support cells for islets/beta cells. Encapsulation alone positively influenced the hMSC secretome compared to monolayer cultures, by promoting the release of anti-inflammatory factors such as the key inflammatory mediator prostaglandin E2 [63]. However, it is uncertain whether the changes in the secretome reflect the 3D environment or the encapsulation material itself. The constant and regulated release of MSC-secreted factors indicates that encapsulation methods should be suitable for the treatment of metabolic disorders such as diabetes [56]. In coencapsulation experiments involving hMSCs and islets in alginate, the viability of the islets did not increase but the SI value increased compared to encapsulated islets alone [64]. These studies showed that encapsulation improves the functionality of hMSCs and beta cells and provides a useful cocultivation strategy in a bioreactor system.

## 3. Bioreactor Technologies for the 3D Cultivation of MSCs

The cultivation of hMSCs in a bioreactor has two main objectives: cell expansion and improved cell functionality. Bioreactor systems are needed to generate enough cells with the required properties, because many cells are required for each treatment (0.4–10·10^6^ MSCs/kg body weight, depending on the disease and type of application [65]). Bioreactors achieve efficient cell expansion by monitoring important parameters such as substrate consumption/metabolite production, cell growth, and differentiation and by allowing the tight control of pH, temperature, and gas supply. MSCs can lose their capacity for self-renewal or differentiate and subsequently lose their multipotency [37, 66–68]. Therefore, the regulation of the microenvironment is a challenging obstacle for bioreactor technologies [33, 61]. In dynamic bioreactor systems, the cellular microenvironment is further influenced by fluid dynamics, which prevent the accumulation of secreted biomolecules, and shear stress can induce either desirable or undesirable forms of differentiation [69, 70]. However, a controlled bioreactor system offers the chance to specifically influence the hMSC secretome and improve the resulting therapeutic effect. An investigation of the impact of dynamic cultivation on the hMSC secretome revealed the upregulation of classical trophic factors such as BDNF, NGF, VEGF, and IGF-1, which were important for the hMSC effect [71]. This highlights the impact of culture conditions on cell functionality. The main conditions required to grow MSCs in bioreactors include a large surface area to volume ratio, a closed system, automated inoculation and harvesting, and the automated online control of culture parameters. Several controlled bioreactor types are suitable for the cultivation of hMSCs, including classical fixed bed, fluidized bed and stirred tank reactors, and alternative systems such as wave reactors, wall-rotating systems, and Vertical-Wheel™ reactors [72]. Moreover, hMSC expansion can also be achieved in spinner flasks. We will not consider spinner flasks, because in our opinion they do not offer the tight environmental control which is needed for 3D cultivation. The most promising bioreactor types for hMSC cultivations are stirred tank reactors (STRs) and fixed-bed reactors (FBRs). These are well characterized and can regulate the cellular microenvironment to ensure the correct proliferation of functional MSCs on microcarriers, in capsules or as agglomerates.

### 3.1. Stirred Tank Reactors for hMSC Cultivation

The cultivation of hMSCs in STRs usually involves microcarriers, but the growth of hMSCs as aggregates or spheroids has also been described. The maximum scale of hMSC expansion reported thus far was achieved in a 50 L STR with a 35 L working volume, resulting in a 50-fold expansion and 2.6·10^10^ cells in total [72]. The expansion of hMSCs is usually a batch-mode process, whereas fed-batch processes are often more advantageous because the process starts with a small inoculum in a low working volume, which increases over time with the simultaneous addition of carrier (for carrier-based processes) leading to higher expansion factors [73]. Working with lower inoculum densities for microcarrier-based processes (100 cells cm^−2^, 5 cells per microcarrier) not only is less expensive but also achieves better hMSC proliferation [74]. In contrast, a low inoculation density does not allow sufficient numbers of intercellular contacts to form when establishing aggregate cultures. An inoculation density of 4.5·10^5^ cells mL^−1^ was recommended [75]. It is often advantageous to allow hMSC inoculums to attach to the microcarrier surface or form aggregates with intermittent agitation or none at all. Agitation is a major process parameter because homogenous mixing is necessary to prevent the formation of substrate gradients or cell-loaded microcarrier/aggregates clumping due to cell bridging. The suspension criteria for agitation can be calculated based on the power input, microcarrier type/cell aggregate properties, and cell growth and can be used for process scale-up [76]. The hMSC expansion process occurs in a three-phase system (medium, microcarrier loaded with cells/cell aggregates, and oxygen gas bubbles). Aeration is often low due to the generally low oxygen demand of mammalian cells (oxygen consumption rates: primary hMSCs 90–100 fmol cell^−1^ h^−1^ [77], hMSC cell line (hMSC-TERT) 300 fmol cell^−1^ h^−1^ [78]). Even so, aeration is an important part of mixing, and it is necessary to optimize hMSC expansion in controlled bioreactor systems in order to understand the mixing characteristics [79]. High aeration and especially agitation ensure proper mixing but induce shear stress. In 3 L microcarrier-based hMSC expansion processes, shear stress is very low (average 0.2·10^−5^ N cm^−2^, maximum 1.2·10^−5^ N cm^−2^ close to the impeller) and does not affect hMSC growth or stem cell fate [79]. Shear stress has a greater impact in agglomerate cultures because it can prevent the formation of stable aggregates and damage the cells, reducing the number of available cells even further [80].

### 3.2. Fixed-Bed Reactors for hMSC Cultivation

In FBRs, hMSCs grow on dense macrocarriers or as larger capsules (500 *μ*m diameter) which form a stable bed inside the reactor. It is challenging to achieve homogeneity and scalability during hMSC expansion in a FBR, although notable improvements have been achieved [81]. The tendency of FBRs to develop channels and gradients in the fixed bed must be addressed during scale-up. However, during hMSC expansion, FBRs are characterized by low constant shear (0.5·10^−5^ N cm^−2^) in the whole reactor space, with no shear peaks near the impeller as seen in an STR. Shear forces are a major problem during the scaling up of a STR because more agitation is needed at larger scales, thus creating smaller Kolmogorov eddies [82]. When the eddy size is ~65% of the microcarrier size, the shear stress can cause significant damage to the cells on the microcarriers. In FBRs, the shear is constant at all scales because superficial velocity stays constant at a value that does not affect hMSC growth (e.g., 1.8 cm min^−1^) [83]. Weber et al. [83] described the expansion of hMSC-TERT cells on a nonporous borosilicate glass microcarrier (diameter 2 mm) in a FBR (300 cm^3^) according to good manufacturing practice (GMP) requirements. The cells could be expanded to 1.6·10^5^ cells cm^−2^ (total 8.7·10^8^ cells) achieving an expansion factor of 29. The use of a nonporous microcarrier facilitates the detachment of cells during harvest and prevents mass transfer limitations. However, the harvest efficiency is a major drawback of the FBR. Even after optimization, a maximum 70% of the expanded cells were harvested with full viability. The viability was reduced by further processing of the harvested hMSCs, for example, during encapsulation, showing that the stress during FBR-harvesting is not acceptable [48]. Similarly, hMSCs have been expanded in a packed-bed reactor at different scales (13–250 cm^3^), resulting in a harvest efficiency of 84%, but only 71% of the harvested cells were viable [84]. A maximum 40-fold expansion factor (1.6·10^8^ cells in total at the 250 cm^3^ scale) was reported, leading to final cell densities of 6·10^4^ cells cm^−2^ (GFP-MSCs) or 1.5·10^4^ cells cm^−2^ (placenta MSCs). A hollow-fiber reactor resulted in an expansion factor of 6 (2.4·10^4^ cells cm^−2^, 3·10^8^ cells total) [85], with production rates of 5·10^7^ cells d^−1^ (for further reading, see [86–88]), whereas a 257 cm^3^ fibrous-bed reactor achieved a 9-fold expansion factor (3·10^3^ cells cm^−2^, 9.2·10^7^ cells total) [89]. Therefore, although hMSCs can be expanded in FBRs, the harvest problem remains to be addressed.

The cultivation of hMSCs in an FBR can also be used for hMSC differentiation or regeneration. Here, nongrowing hMSCs are cultivated in the form of capsules or aggregates to improve cell viability or functionality. This can be set as a second process following the expansion step to regenerate cells, which suffer during the harvest and encapsulation procedures. For example, 2000–3000 nonproliferating hMSC-TERT cells were encapsulated in an alginate double layer, generating capsules with a core diameter of ~400 *μ*m and an overall diameter of 500–600 *μ*m. The capsules were cultivated as a fixed bed in a single-use syringe in a perfusion arrangement. The vitality and also the quality of the cells increased over the duration of cultivation, whereas the cell number declined. This may reflect the degradation of necrotic cells, which suffered during the harvest, encapsulation, or freezing procedures. For some differentiation setups, for example, osteogenic differentiation of hMSCs, scaffolds mimicking the *in vivo* structures can be 3D-printed and used in a fixed-bed reactor. This gives an additional differentiation stimulus to the cells as they respond to surface stiffness and structure [90, 91].

## 4. Bioreactor Technologies for 3D Beta Cell Approaches

Compared to hMSCs, where bioreactor cultivation has become very common, beta cells are rarely cultivated in bioreactors. Currently, the mass production of beta cell *in vitro* is challenging and has not succeeded in the way researchers hoped. In standard 2D cultures, beta cells lose their functionalities, including the ability to secrete insulin in a glucose-dependent manner. This can be addressed by 3D cultures because the 3D cell-cell contacts preserve the beta cell phenotype, improve the regulation of insulin gene expression [92, 93], achieve higher cell viability, and increase insulin secretion after glucose stimulation [92, 94–96]. But even in 3D cultures, beta cells tend to grow extremely slow. Growth rates (*μ*) for porcine islets varied from 0.17 to 0.27 d^−1^, which is a population-doubling time (*t*_D_) of up to 100 h [97]. Engineered beta cell lines may be suitable because they often grow much more quickly than wild-type cells (e.g., 1.1B4 cells: *μ* 0.84 d^−1^, *t*_D_ 20 h), but this is only possible if the engineered cells show the desired properties. Beta cells also consume a lot of oxygen, but if we compare the oxygen consumption rates of beta cells (primary cell: 23 fmol cell^−1^ h^−1^, max. *q*_ox_ 139 fmol cell^−1^ h^−1^ [98]; bTC3: 114 fmol cell^−1^ h^−1^, MIA PaCa-2: 108 fmol cell^−1^ h^−1^ [99]) or islets (230 fmol cell^−1^ h^−1^ [100]) with other cells (*q*_ox_ from 3.6 to 1260 fmol cell^−1^ h^−1^ [99], e.g., hepatocytes 324 fmol cell^−1^ h^−1^, adipocytes 430 fmol cell^−1^ h^−1^), the rates are not excessive. It is clear that beta cells suffer from hypoxia and die if their oxygen demands are not met, but other obligate aerobic cells behave in the same manner, so this is not an exclusive property of beta cells. It is interesting that the oxygen demand of beta cells is coupled to their insulin secretion and can be increased by adding glucose to the medium. Under basal conditions (5.8 mM glucose) the *q*_ox_ of human islets was 1.5·10^4^ fmol IE^−1^ h^−1^, which is less than half the value in the presence of 33 mM glucose (*q*_ox_ = 3.6·10^4^ fmol IE^−1^ h^−1^). Insulin secretion increased in proportion with the glucose concentration in this setup [101]. To summarize, the cellular microenvironment and nutrient supply for beta cells must be tightly controlled, maybe even tighter than those for hMSCs. This can only be achieved in controlled bioreactor systems. However, cell expansion in bioreactors is uncommon for beta cells. The few bioreactor concepts that have been developed are mainly for tissue engineering or diabetes models. These systems are often very small, that is, down to microreactor scales of a few milliliters. Microreactors in a microtiter well format can be suitable for drug screening [102], but larger bioreactor concepts are needed for the high-cell-mass expansion and cultivation of beta cells as discussed below.

### 4.1. Rotating Wall Vessel Reactor for Beta Cell Cultivation

The rotating wall vessel reactor (RWVR) has been used for the cultivation of human and other mammalian islets in aggregate culture. The rotating wall applies low shear stress, but there is proper mixing which ensures efficient nutrient and oxygen transfer within the medium. The islets are suspended by microgravity, which is achieved by the continuous rotation of the medium. A drawback of this reactor type is the clumping of the islets, which has been addressed by scaffold culture [103]. The functionality of bioreactor-cultivated murine islets improves as shown by the higher SI value compared to freshly isolated islets. The bioreactor-cultivated islets also developed unique and multiple nutritional channels [104]. Human islets (50–150 IE mL^−1^) cultivated in a RWVR (10 mL, 30°C) showed a stable islet structure and higher SI values compared to 2D static cultures. Dispersed islet cells reaggregated within the reactor [105]. A murine pancreatic cell line (b-TC-6) cultivated on the microcarrier Cytodex-3 in the RWVR proliferated over 12 days with improved insulin gene expression and a clear response to glucose-stimulation [106]. The murine pancreatic cell line MIN6 was used to form spheroids in a RWVR. The optimal seeding density was 6·10^6^ cells mL^−1^, because with increasing seeding densities (2·10^5^ to 6·10^6^ cell mL^−1^) the number of spheroids increased (14 to 1100) but the average spheroid diameter (600 to 220 *μ*m) and cells per spheroid (184·10^3^ to 9·10^3^) decreased. The spheroids formed in the RWVR showed higher SI values than 2D-cultured MIN6 cells, the shape and size of spheroid cells was similar to pancreatic islets, and the expression of the genes *insulin2*, *glucokinase*, *SETD1A*, and *Kir6.2* was stronger than in MIN6 cells from 2D cultures [92]. Therefore, the RWVR helps to improve the functionality of islets and beta cell aggregates in vitro and could also be used for beta cell expansion. However, most published data reflect small-scale experiments which would not satisfy the needs of cell therapy.

### 4.2. Stirred-Tank and Fixed-Bed Reactors for Beta Cell Cultivation

Like hMSCs, beta cells can be cultivated in classical bioreactor systems which have well-characterized scale-up parameters. In contrast to hMSCs, beta cells are cultivated in aggregates or capsules rather than on microcarriers. Beta cell expansion using microcarriers in an STR was carried out using trypsin-dispersed human pancreatic islet cells grown on 1 g L^−1^ Cytodex-3 microcarrier. With a 1.2 L working volume, the cell number was doubled, reaching 3.1·10^5^ cells cm^−2^ (total 1·10^8^ cells). Cell growth was very slow (*μ* = 0.11 d^−1^), but the cells were functional in that they secreted insulin at a rate of 4.5·10^3^ fg cell^−1^ [52]. The pancreatic cell line BRIN-BD11 was expanded in a STR with a 1 L working volume on Hillex or PlasticPlus microcarriers (both SoloHill), achieving an expansion factor of 2.8. Compared to 2D cultures, cells grow faster in bioreactors (*μ* = 0.49–0.52 d^−1^; *t*_D_ 31 to 34 h). Cells expanded on PlasticPlus microcarriers produced 2.6-fold more insulin than Hillex and 2D cultures. This provides another excellent example of how the growth surface material and its properties might influence the behavior of beta cells [107]. We found no data for beta cell aggregate/islet cultivation in an STR although several publications discussed beta cell cultivation in spinner flasks but described this as stirred bioreactor cultivation. As stated above, we do not agree with this classification because spinner flasks lack any form of environmental control and are unsuitable for large-scale expansion. Even so, because of the missing data for beta cell aggregates in STRs, we will review helpful results from spinner cultivations. Porcine pancreatic cells were seeded in spinner flasks in different densities (6.3·10^3^, 5·10^4^, and 1.3·10^5^ cells mL^−1^). The high-density culture achieved a ninefold increase in the number of insulin-releasing cells, and many glucose-responsive spheroids were formed after 9 days [108]. MIN6 cells formed aggregates in spinner flasks with their diameter increasing from 100 to 800 *μ*m over time. With an aggregate size > 200 *μ*m, cell viability decreased probably due to mass transport limitations. An expansion factor of 14 was achieved with slightly longer growth rates (*t*_D_ 87 h) compared to 2D culture (*t*_D_ 72 h). The pseudoislets formed in spinner flasks had lots of the structures of native islets, whereas glucose-dependent insulin release was not significantly improved compared to 2D cultures [109].

Fixed-bed systems have also been used to cultivate beta cells. Porcine pancreatic cells were grown in an alginate-filled hollow fiber FBR where the single cells formed aggregates. The cells remained viable and secreted more insulin compared to an aggregate suspension culture [110]. Hollow fiber FBR has been used to expand rat insulinoma cells fourfold, reaching a final density of 5.7·10^4^ cell cm^−2^ (1.3·10^8^ cells in total) [111]. Nothing has been reported about the harvest of beta cells from FBRs, but it can be assumed that the same problems that apply to hMSCs were encountered. STRs or FBR using microcarriers or aggregates seemed to be suitable for beta cell expansion and cultivation, although more basic work is needed to understand these cell changes even in classical bioreactors.

### 4.3. Other Reactor Types Used for Beta Cells

An interesting alternative to the systems presented above is the fluidized-bed reactor. In a brand new study, alginate-encapsulated MIN6 cells were cultivated in a small fluidized-bed reactor (15 cm^3^). The fluidization point was determined, and full fluidization occurred at a superficial velocity of 1.13 cm min^−1^. The cells within the capsules (diameter 180–220 *μ*m) were viable over 7 days and showed a higher SI value (3.7) in the fluidized-bed reactor compared to static cultivation (SI = 2.3) [112]. Certain other bioreactor types may be suitable for beta cell cultivation, including wave reactor systems, but none of these has yet been described for this purpose.

In summary, few studies have considered beta cell/islet cultivation in bioreactors. One can argue that beta cell expansion can be solved by the expansion and subsequent pancreatic differentiation of pluripotent iPSCs. However, this does not address the issue of maintaining these cells in a functional state *in vitro*. The persistence of beta cell function *in vitro* requires an environment that is sufficiently similar to the *in vivo* context. Islets *per se* satisfy of these requirements such as the provision of an ECM and supporting cell types such as endothelial and mesenchymal cells carried over from islet isolation, whereas iPSCs do not. In the human body, islets are surrounded by a milieu of ECM and mesenchymal, endothelial, neuronal, and exocrine cells, many of which act to support beta cell identity and functionality [113]. Current culture conditions must be modified to maintain functional beta cells, and within bioreactors, optimal 3D environments can be created. Furthermore, rather than cultivating beta cells in isolation, cocultivation with other cell types, for example, supporting hMSCs, might bring more success.

## 5. Engineering of Bioreactor Cocultivation with MSCs and Beta Cells

Cocultivation at larger scales is needed to produce an appropriate number of functional beta cells for cell therapy or for large-scale drug screening. Large-scale cocultures in bioreactors have only rarely been reported for mammalian cells. At such large scales, the problem of heterogeneity arises, which can lead to instability within the bioreactor and a loss of cell viability, so a well-balanced and tightly controlled culture environment is needed at larger scales to stabilize the complexity of the coculture. Before cocultivation is possible in a chosen bioreactor system, preliminary investigations are needed to define the coculture mode (direct, indirect, or mixed). This can be achieved at smaller scales, for example, in transwell plates or microreactors. Because secreted factors are important for hMSC and beta cell cocultivation, the hydrodynamics in bioreactors, which influence the distribution of the secreted molecules, should be considered at an early stage [114]. Furthermore, the cocultivation ratios of the cells need to be determined. Depending on the process setup and the growth rates of the cells, the population sizes in a coculture often differ vastly, with one being much more dominant. For hMSCs and beta cells, hMSC outcompete beta cells because their growth rate is up to three times faster (worst case scenario: *μ* (hMSC) = 0.5 d^−1^, *μ* (beta cells) = 0.17 d^−1^). To reach a stable population, or even better a coculture with dominant beta cells, the ratios and culture conditions have to be optimized. However, the growth rates of cocultivated hMSCs and beta cells may differ from the pure culture growth rates because the cells might influence each other's proliferation, as shown for hMSCs cocultured with human umbilical vein endothelial cells (HUVECs) [115]. The ratio can also influence the therapeutic effect of the cells and their behavior *in vivo*.

### 5.1. Important Parameters for hMSCs and Beta Cells

Cocultured hMSCs and beta cells are biologically very different, but from a bioprocessing perspective, they have similar characteristics. Biologically, hMSCs are mesoderm-derived multipotent cells with a high differentiation capacity, whereas beta cells are fully differentiated, unipotent cells derived from the endoderm. From the bioprocessing perspective, both cell types grow as adherent cells within the same temperature range (30–37°C) and pH range (7.0–7.4), and they consume the same nutrients (key metabolite = glucose) and oxygen at similar rates (Table 1). Because islets consist mainly of beta cells (a single islet contains 1560 cells, of which 1140 are beta cells [116]), the properties of beta cells are primarily responsible for islet behavior in bioreactors. The growth rates of hMSCs and beta cells can differ significantly as stated above, so it can be useful to establish an optimal environment for beta cells in the cocultivation setup, thus slowing the growth of hMSCs to a desired level. However, the hMSCs must still secrete all the trophic factors needed to support the beta cells. The behavior of both cell types in coculture is difficult to predict because they influence each other in unknown ways. Saleh et al. [117] show the positive effect of cocultures of hMSCs and HUVECs compared to cocultures of hMSCs and adult human dermal fibroblasts (HDF). The HUVEC/MSC cocultures formed hybrid spheroids comprising elongated and flattened endothelial-like cells on the outer layer whereas such cells did not form in the HDF/MSC spheroids. A clear boundary between cell morphologies indicates the self-arrangement of the cell populations, which was confirmed by transmission electron microscopy. The HUVEC/MSC spheroids showed enhanced osteogenic differentiation compared to the HDF/MSC spheroids, and the reverse effect was shown for adipogenic differentiation. This study therefore suggested that somatic cells promote the self-assembly, differentiation, and activity of hMSCs. All these parameters must be considered and balanced in a cocultivation setup.

### 5.2. Suitable Cocultivation Concepts for hMSCs and Beta Cells in Bioreactor Systems

New bioreactor systems are not necessary for the cocultivation of hMSCs and beta cells because the classical systems appear to be suitable. However, it might be useful to separate cell expansion from cocultivation; that is, first expand the pure cultures to generate the cells needed for the coculture and then combine them to improve the function of beta cells in a second process step. For the expansion process, it can be sufficient to improve the growth of beta cells using conditioned medium from hMSCs. Carrier-based expansion in a STR or FBR is suitable for the expansion process, because new resolvable carriers will soon be available commercially to address the harvest problem in these processes. Here, the cells are not trypsinized, but the carrier material is degraded to harvest the cells, thus preserving the surface proteins of the cells to improve their functionality and reduce the stress during harvest. After expansion, the functionality of the beta cells can be improved by cocultivation. Alternatively, the expansion and functionalization of beta cells can also be combined in one process step. Process engineering is a challenging part of the development of a cocultivation strategy. Various aspects need to be considered; for example, processes must fulfill the demands of GMP and process analytical technology (PAT) and the culture medium must satisfy the needs of both cell lines. It is a matter of balancing the demands of two cell types in a system with many unknown variables. In our opinion, hMSCs are more robust *in vitro* than beta cells, so the latter should be favored when setting the process parameters. Certain cocultivation bioreactor concepts may be suitable depending on the cocultivation mode (Figure 2). For direct cocultivation, STRs, FBRs, and fluidized-bed reactors may be appropriate, in batch or even better in fed-batch or perfusion mode. The beta cells and hMSCs can be cultivated as mixed aggregates, capsules, or on carriers. Indirect cocultivation offers more options, including elegant approaches such as using the encapsulated beta cell aggregates as carriers for the hMSCs. In the indirect cocultivation approach, it is also possible to create a cell-specific optimal microenvironment for each cell type, for example, by compartmentalizing them with membranes. Future work will focus on realizing these cocultivation bioreactor concepts for hMSCs and beta cells and testing which concepts achieve the best results.

## 6. Outlook and Conclusions

In most cases, hMSCs are used alone for cell therapy, but more recently, hMSCs have been considered as *in vivo* supporter cells for other cell types, including beta cells. One major problem with these treatments is that a large proportion of the hMSCs die at the transplantation site. The hMSCs are not able to achieve their full potential, which reduces the therapeutic efficacy. Beta cells also die at the transplantation site, but a combination of hMSCs and beta cells has been shown to support greater survival. Furthermore, the hMSCs can strengthen the beta cells and improve their functionality *in vitro* when the cells are cocultivated. Here, it remains unclear which cocultivation mode is the best and which hMSC type is most suitable. Because hMSCs can be isolated from different sources, for example, bone marrow, adipose tissue, and umbilical cord, their properties are distinct. Umbilical cord hMSCs are very young and primitive and therefore offer advantages in a direct cocultivation mode. They can differentiate into islet-like clusters and thereby promote beta cell functionality. Although the hMSC types share many secreted factors in common, their secretome differs in terms of the concentration of these factors as well as other factors which are only secreted by certain hMSC types [118]. Based on the type-specific secretome, specific hMSC types are likely to improve the functionality of different cells in indirect cocultivation approaches. The hMSC type which is best to restore the functionality of beta cells is unknown. It is difficult to generate large numbers of medical grade hMSCs and functional beta cells for therapy, and still little is known about the behavior of cells in 3D structures; thus, new 3D cocultivation concepts are required. Few researchers have focused on cocultivation concepts for mammalian cells in liter-scale bioreactors, and no such cultivation strategies have been reported for hMSCs and beta cells or other somatic cells. These production scales are necessary for cell therapy and even for large-scale drug screening. Therefore, it is time to concentrate on the open bioengineering questions for hMSCs in coculture with beta cells and other somatic cells in bioreactors. Otherwise, the groundbreaking efforts made in basic research cannot be brought into clinical practice without a significant delay. The 422 million diabetes patients are waiting for an option that cures their disease, but without bioengineering and suitable 3D bioreactor concepts, beta cell therapy cannot succeed.

## Figures and Tables

**Table 1:** Process-relevant parameters for human mesenchymal stem/stromal cells (hMSCs) and islets or beta cells based on our own data and the literature.

		hMSC	hMSC-TERT	Islet	1.1B4	MIN6^∗^
Biological parameter	*μ* [d^−1^]	0.33–1.1	0.4–0.7^#^	0.11 [49]	0.84	0.19–0.23 [100]
	*t*_D_ [h]	15–50	24–42	151	20	72–88
	*q*_GLC_ [pmol cell^−1^ h^−1^]	0.13–0.7	0.02–0.5^#^	n.d.	0.08^#^	n.d.
	*q*_ox_ [fmol cell^−1^ h^−1^]	90–100 [77]	300 [74]	23–139 [89]	n.d.	

Process parameter	Seeding density [cells cm^−2^]	1000–10,000^#^		Depends on reaggregation method	5000–10,000^#^	>80,000
	Final cell density [cells cm^−2^]	5·10^4^ (dynamic)^#^ 2·10^5^ (static)^#^			1·10^5^ (static)^#^	2-3·10^5^ (static)^#^
	Max. working volume [L]	35	2.4^#^	<0.1		
	pH [−]	7.0–7.4				
	*T* [°C]	30–37				
	*k*_L_a [h^−1^]^+^	0.6	4.4	0.2	n.d.	
	Shear sensitivity *τ* [N cm^−2^]	1.2·10^−5^		Mostly static cultivation with low shear		
	Growth surface material	TC-treated polystyrene, micro- and macrocarrier with different coatings		Mainly TC-treated culture vessels (polystyrene)		
	Aggregate formation	Possible, but lower tendency		Strong tendency		
	Culture media	SCM, SFM, CDM		SCM with 10–20% serum		
	Glucose concentration [mM]	Usually 5.5, 5–25		5.8 recommend, 5–25		

Abbreviations: *μ*: growth rate; *t*_D_: doubling time; *q*_GLC_: glucose consumption; *q*_ox_: oxygen consumption; *k*_L_a: oxygen transfer coefficient; TC: tissue culture; SCM: serum-containing medium; SFM: serum-free medium; CDM: chemically defined medium; n.d.: not determined. ^∗^Murine origin; ^#^own data; ^+^aeration with air.
